# Some of the Leading Men and Events in the History of American Medicine1Presidential address to the Alumni Association, Medical Department University of Buffalo, at its thirty-first annual meeting, May 29, 1906.

**Published:** 1906-08

**Authors:** Fridolin Thoma

**Affiliations:** Buffalo, N. Y.


### Some of the Leading Men and Events in the History of American Medicine.[1]

By FRIDOLIN THOMA, Buffalo, N. Y.

THE first physician or surgeon of whom we have an authentic record of having come to America was one John Wolton, or Wooten, who came to Jamestown in 1607 with Captain John Smith—a man no doubt of high heart and vigor, a worthy predecessor of that long line of resourceful and able men who have honored American medicine. Besides Wolton, the surgeon, other doctors came to these shores about the same time, one being Dr. Walter Russell, perhaps a man of science and holding a degree. In the same year, 1608, Anthony Bagnall, chirurgeon, sailed with Captain Smith upon the Chesapeake, but of him, Russell and Wolton we know little.

In 1610 came Lawrence Bohun and in 1624 John Pott, who was temporary Governor in 1628. In 1629 the early Virginia doctors went before the legislature to obtain laws regulating medical practice. Throughout the colonies quackery flourished with little let or hinderance. The great distances, the infrequent needs of the people, the loose methods and credulity of most of the regularly licensed physicians, gave great opportunities to irregular practitioners.

The first law, passed on October 21, 1639, was "an act to compel physicians and surgeons to declare on oath the value of their

1. Presidential Address to the Alumni Association, Medieal Department University of Buffalo, at its thirty-first annual meeting, May 29, 1906.

medicines." In 1620 Samuel Fuller came to Plymouth; he was the first physician in New England and for some time the only one, and he brought a small stock of drugs with him. The first winter in Plymouth needs no telling here, but we must believe that there was enough practice for Deacon Fuller and his faithful wife. Already grown and matured he practised thirteen years and then died. His wife was taught his work in a rough way, to act as a midwife, and both together served their neighbors well.

John Winthrop, Jr., was born in 1605 and became Governor of the New Haven Colony in 1657, a contemporary of Harvey and probably studied with that master before coming here, being no doubt of good repute in science for he became a founder of the Royal Society. It is hard to say what may have been the medical value and service of Winthrop to the colonists, but it would seem that, like Endicott and Winslow, the latter of whom is also reckoned among the doctors, he was rather a board of health and general supervisor than a constant, active practitioner.

One cannot tell the story of American Medicine as a whole because there was no whole. There were, however, four principal medical centers before the revolution, and if we count Virginia and Maryland there were six; but the principal centers were naturally the leading seaboard cities,—New York, Philadelphia, Boston, and Charleston.

In 1637 came M. La Montagne, the first New York physician, and as of most of those early physicians we know little of him. He wrote nothing medical of moment but followed the best practice of his time. He was described as learned and skilful. La Montagne was, however, not alone in Manhattan for, the year after his coming, there arrived Gerret Schult and Hans Kierstede, surgeons. Then there was Samuel Megapolensis who was born in this country and graduated in 1657 an M.D. of Utrecht. There were a few others,—Abraham Staats, Vanevenger L'Orange, J. Hughes, Du Park and Johannes Kerfbyle, who is on record as having made a medico-legal autopsy.

In 1682 a Welshman, Thomas Wynne, came to Philadelphia, who it is said was the most thoroughly equipped and learned physician that until then had visited America. Most of these early Philadelphia physicians were Welshmen and many were kinsmen. Two of the most notable advances in the science of medicine which have been made in this country originated in Boston; the first was the introduction of inoculation for smallpox; the other the introduction of ether anesthesia, and both received their initial impulse from men not strictly within the ranks of physicians.

June 21, 1721, must be marked as the day of greatest import in the history of American Medicine up to that time, and very few days equal it since, when Zabdiel Boylston inoculated his thirteen-year-old son and his two negro servants for smallpox. Dr. Boylston was born in Brookline, Mass., in 1635. His father was Thomas Boylston, an Englishman with an M.D. degree from Oxford. He studied with his father and also with John Cutter, a man of some local reputation. It does not appear that he received the doctor's degree but he was the first native American who was made a member of the Royal Society.

Cadwallader Colden who came to Philadelphia in 1710, was graduated an M.D. from Edinburgh when twenty years old. After a residence here of five years he went to London to improve his knowledge; in 1716 he returned to America and in 1718 he took up a permanent residence in New York. He was noted for his prolific writings, and also held many public offices—Lieutenant Governor, Surveyor General of the Province, Master in Chancery and a member of the Governor's Council.

Thomas Cadwallader was born in Philadelphia, the son of a prominent physician, well educated for his time and perhaps more noted for his broad humanities. He studied under Cheselden and on his return from London established by request a class in practical dissections, demonstrating anatomy as well as performing autopsies for his less well-informed colleagues. In the same year, 1750, a criminal named Hermanus Carroll was executed for murder in New York City and his body was dissected by John Bard and Peter Middleton, for the instruction of the young men then engaged in the study of medicine. This is the first essay made in the colonies for the purpose of acquiring medical knowledge of which we have any record.

While the eastern and middle colonies were producing their numerous practitioners and a few better known to us, South Carolina was honored by a group of five men who deserve special notice. They were Chalmers, Linnig, Garden, Moulton, and Bull; not that their contributions were of great permanent value, but because they were men of sound understanding and unusual cultivation, who seemed to have appreciated that progress is not only attained by the brilliant discoveries of the few but by the obscure toil of the many.

Thomas Bond was born in 1712 in Maryland, was in comfortable circumstances and received an excellent education. He was not graduated in arts but early began his medical studies in the office of a Dr. Hamilton and, after having passed some six years under his tutelage, he went to Europe for a broader learning. Bond came home in 1734 and settled in Philadelphia. In 1751 he launched his great project of establishing a municipal hospital. He enlisted the aid of that vigorous philosopher, Benjamin Franklin, who at once saw the value of the proposal and put it before the community. After the sum of 2,000 pounds had been raised by public subscription and another 2,000 pounds added by the state legislature of Pennsylvania, the design was carried into execution. A house was rented on the south side of Market Street (then High) below Seventh and was ready for patients by February, 1752. Rules were adopted and signed by Benjamin Franklin, clerk, by the Speaker of the Assembly and by the Attorney General. The first staff consisted of Thomas Bond, Lloyd Zachary, and Phineas Bond, whose services were rendered gratis. There was also a consulting staff composed of Cadwallader, Graeme Moore and Redman. So the Pennsylvania Hospital started out humbly enough but in competent hands, and after four years of such existence the corner stone of the present ancient building was laid, Benjamin Franklin writing the inscription:

In the year of Christ
1755
George the Second Happily Reigning
(For he sought the happiness of his people)
Philadelphia flourishing,
(For its inhabitants were public spirited,)
This Building
By the bounty of the Government and of many private persons
Was piously founded
For the relief of the sick and the miserable.
May the God of Mercies bless the undertaking.

The first three great names connected with its first half century are those of Franklin, Bond and Rush, and it continues today a splendid monument to their wisdom, foresight and ability. Benjamin Rush has been called the father of American medicine, but we must call John Morgan its grandfather. John Morgan was born in Philadelphia, in 1736, and received the degree A.B. from the College of Philadelphia in 1757 with the first class that graduated from that institution. He studied medicine with Dr. Redman and served in the war against the French. In 1760 he resigned from the army and went to Europe to pursue his medical studies. He passed several months with William Hunter, studying anatomy and surgery, then went to Edinburgh where he became the friend and pupil of Cullen, the Munroes, Rutherford, Wyth and Hope, where he spent two years and was graduated a doctor of medicine from the University. Thence he repaired to Paris, studied under Sue and was admitted to the Academy of Surgery. He also traveled to Holland and Italy where he won the friendshp of Morgagni. After all his traveling he returned to London where he was made a Fellow of the Royal Society, as well as an M.C.P. of Edinburgh. During his travels he conceived the idea of forming a medical school and upon his return to America he set to work to establish one, in which project he was ably seconded and assisted by his friend and townsman, William Shippen, Jr. On the 30th day of May, 1765, Dr. Morgan delivered his "Discourse upon the Institution of Medical Schools in America." He had written this when in Paris and it had undergone a careful scrutiny by Dr. Fothergil, Dr. Hunter and Dr. Watson of London. In it he recommended a very comprehensive preliminary education preparatory to the study of medicine.

It would not be fair to assume that Morgan was the only promoter of the scheme; mention has already been made of William Shippen, Jr. The two were born in the same year, 1736. Shippen came of a well-known medical family, his father being William Shippen, Sr., who was a prominent practitioner of Philadelphia. He enjoyed a liberal education, was graduated from the College of New Jersey, as Princeton was then called, and after studying some time with his father he, too, went to Europe and enjoyed the instructions of John Hunter in Anatomy and William Hunter and McKenzie in midwifery. He received his degree of M.D. from the University of Edinburgh in 1761, returned to this country in 1762, and in March of the same year began the delivery of a series of lectures on midwifery, the best special course ever given in this country up to that time.

William Hunter, of Rhode Island, a relative of the famous Scotch Hunters, and other energetic men had done similar work, useful though spasmodic. Thus it came about that in 1765 these two young men, then but 29 years old, approached the great subject with almost equal preparation, vigor and enthusiasm. Their fitness was so apparent that the trustees of the college arranged to launch the medical school. Morgan and Shippen were at once chosen for the two important chairs of theory and practice of medicine, and of anatomy and surgery respectively. These two men controlled for many years the policy of the school. The college was to confer two degrees in medicine, the bachelor's and the doctor's, although in the next generation the bachelor's degree was abandoned. Two other men were now appointed to the little staff —Adam Kuhn, professor of materia medica and botany, and Benjamin Rush, professor of chemistry. Adam Kuhn was born in Philadelphia in 1743, first studied medicine with his father, and in 1761 went abroad and studied medicine at the University of Upsul in Sweden, where he also pursued the science of botany under the great Linneaus. Subsequently Kuhn studied medicine in London, finally going to Edinburgh, from which university he received the degree of M.D. in 1767. He returned to America in 1768, lectured on botany in May of that year, and lectured for twenty years on materia medica.

Benjamin Rush was born on his father's farm a few miles from Philadelphia in 1745. He obtained the degree of A.B. from Princeton in 1760, before he was fifteen years old, and at once began the study of medicine, serving as an apprentice to John Redman. He went to Edinburgh in 1766 and received from that university the degree of M.D. in 1768, returned to Philadelphia in August, 1769, and was elected to the chair of chemistry. In 1771 the degree of M.D. was conferred upon Jonathan Elmer, Jonathan Potts, James Tilton and Nicholas Way.

In 1768 the medical school of Kings College was established in the city of New York. The chairs were held by Samuel Clossy, anatomy; John Jones, surgery; Peter Middleton, physiology and pathology; James Smith, materia medica and chemistry; John V. B. Tennant, midwifery; and Samuel Bard, theory and practice of medicine. This school was the first to confer the M.D. degree in this country—namely, in 1770, upon Robert Tucker, and in May, 1771, upon Samuel Kissam.

In connection with the founding of these two schools it is interesting to note that Morgan and Middleton, the representative initial spokesmen, placed the highest value upon a broad preliminary education, an equipment which we now, after a lapse of more than a century and a quarter, are beginning to demand of the matriculates in our leading schools.

In 1807 the New York University started a school of its own and gave it the name now famous,—the College of Physicians and Surgeons,—but the new institution soon got into trouble, dissentions in the faculty soon brought it to a low ebb and the lack of consistent courses so discouraged the students that there was a great falling off in their numbers. The fact was that there was not enough clinical material nor teachers in New York at that time. The only remedy lay in a union and that was accomplished in 1811. Samuel Bard was made president and an efficient and congenial faculty was selected from the rival staffs and the College of Physicians and Surgeons started out on its long and successful career.

In New York the medical college preceded the hospital and to supply the lack Samuel Bard made such an eloquent plea for the importance and necessity of a hospital that it was at once taken in hand by the citizens, Governor Sir Henry Moore heading the list and urging it upon the legislature. Dr. Fothergil, of London, with the aid of Sir William Duncan, also raised considerable money.

In 1770 Drs. Bard, Jones and Middleton petitioned their friend Cadwallader Colden, who was then Lieutenant Governor, to further this great object of his professional colleagues, and he granted a charter of incorporation under the title "The Society of the Hospital of the City of New York," and early in 1775 a fine stone building was practically completed, when it took fire and burned to the ground. Again more money was raised and another building was built, and the first use it was put to was the holding of the Provincial Congress in it in 1776.

In 1783 the Harvard Medical School was started on its long and honorable career by John Warren, professor of anatomy and surgery; Aaron Dexter, professor of materia medica and chemistry, and Benjamin Waterhouse, professor of theory and practice of medicine. The lectures were given in Cambridge in the college buildings until 1810, when the medical school was transferred to Boston. The first student upon whom the M.D. degree was conferred was John Fleet in 1788, and in 1789 there were two graduates, Peter DeSales Lattereire and William Pearsons, and in 1790, Nathan Smith.

In 1798 the medical department of Dartmouth College was established largely through the efforts of Nathan Smith, who was for many years its only professor. Eighty years ago he was a tower of strength for New England. He did an ovariotomy, twelve years after McDowell, but he was ignorant of his predecessor.

The first association of physicians into a society of which there is any record in America, was in Boston. It existed from 1735 until at least 1741, when it disappeared in the sands of time. The oldest of still existing medical societies is The Medical Society of New Jersey. The first of their meetings was held at a Mr. Duff's house in the city of New Brunswick on Wednesday, July 23, 1766, at which time and place the constitution and regulations were adopted.

The Medical Society of the County of New York was established in 1794 and continued in existence until 1806, when it became the Medical Society of the State of New York.

According to the Universal Asylum and Columbian Magazine for April, 1790, we find that the American Medical Society was established in the year 1773 with the following officers:

President, William Shippen; Vice-president, William B. Duffield; treasurer and perpetual secretary, Henry Stuber; annual secretary, John Baldwin. The first American medical periodical was published by Elihu Hubbard Smith,—"The New York Medical Repository."

In the Centennial year of 1876 there was published in Philadelphia a little work entitled "A Century of American Medicine." The essay on surgery was written by Samuel D. Gross and in it he says:

"Finally, let it not be supposed from what precedes that the American surgeon is a mere operator; if he ranks high in this particular he ranks high also as a therapeutist. Nowhere, it may be safely asserted, are the great principles of surgery better taught or better understood than they are in this country. As a general practitioner, skilled in diagnosis, and in the art of prescribing, it is no presumption to affirm that he has no superior. If as a body we are deficient in any particular, it is in the more refined and subtle portions of our studies, studies which are after all of no essential practical importance, and which, it is not too much to say, will in due time receive their just proportions."

If, eighty years ago, one had asked the name of the best known and greatest living American surgeon, the reply would have been Dr. Physick. Phillip Syng Physick was born in Philadelphia July 7, 1768. and died there on December 15, 1837. He was graduated a bachelor of arts from the University of the State of Pennsylvania in 1785 at the age of seventeen. He was a student in Adam Kuhn's office, and in 1789 his father took him to London, where he had the good fortune to be taken into the family of John Hunter. He was made house surgeon at St. George's Hospital; he also studied in Edinburgh, where he received the M.D. degree in 1792, returning to America in the same year. In 1794 he was elected surgeon to the Pennsylvania Hospital. In 1800 he was asked to lecture on surgery to certain students in the University school, and became professor of surgery in 1805. Physick was professor of surgery for thirteen years. His lectures were marked for sound statements, concise observations, and conclusions so inevitable that a great impression was made. He was widely famous as a brilliant hospital operator and clinician and also was an able mechanic in orthopedic surgery. But it was lithotomy that gave him his greatest fame. In 1818 his nephew, John Syng Dorsey, died. He had been professor of anatomy and was most successful. After his nephew's death, Physick was persuaded to give up teaching surgery and to take the chair of anatomy, in which he was but a feeble reed. He clung to the uncongenial work for twelve years before he resigned. He died, as stated before, December 15, 1837, and was buried as the "Father of American Surgery."

From Physick we turn at once to a man of whom most truly it must be said that he was not without honor, save in his own generation,—Ephraim McDowell. Ephraim McDowell was born in Rockbridge county, Virginia, November 11, 1771. In 1782

his father, Samuel McDowell, was appointed as a commissioner to adjust the western land grants allotted by Virginia to its veterans, out of its Kentucky territory, and he settled in Danville, was appointed judge of the District Court and lived there until his death in 1817, after his son had done his greatest work. It was in that vigorous frontier life that young McDowell grew up, getting what rambling education was to be found in the classical seminary of Messrs. Worley and James, who migrated between Bardstown and Georgetown. He did not settle down to serious work until he was nearly a man grown. When twenty years old he decided to study some profession and seeing the opportunity of a good doctor in his own rapidly growing state decided on medicine. There being no competent preceptor near Danville he went back to his old home in Virginia and entered the family of a Dr. Humphreys in Staunton, about thirty miles from his native place. He stayed there two years when his father sent him to Edinburgh. Gregory, Black and Munro the second were the great men then, but John Bell was his hero. Bell was not as yet giving his lectures on surgery at the university, but McDowell took one of his private courses and rejoiced in the fact the rest of his life. In 1795 he had to return home without completing his course and without the doctorate degree. He settled in Danville at once and lived there ever after, from the outset well-nigh swamped with a great burden of practice.

In 1809, when thirty-eight years old and fourteen years in practice, McDowell performed ovariotomy. It had never been done before and for centuries surgeons had regarded it as impossible, but out of the American wilderness came the pioneer to show the way. In the autumn of 1809 McDowell was consulted by a Mrs. Crawford, the subject of a large ovarian cyst. Mrs. Crawford drove sixty miles to McDowell's and was operated upon there. No preparation of the patient was made beyond giving her a large dose of opium. The account runs thus: "The patient being on the table I marked with a pen the course of the incision to be made, desiring him (nephew James) to make the external opening, which in part he did. I then took the knife and completed the operation as stated in the Medical Repository. Although the termination of this case was most flattering, yet I was more ready to attribue it to accident than to any skill or judgment of my own, but it emboldened me to undertake similar cases and not until I had operated three times, all of which were successful, did I publish anything on this subject. I then thought it due to my own reputation and to suffering humanity to throw all the light which I possessed upon the diseased ovaria."

He did thirteen ovariotomies in all, eight of which recovered. McDowell performed numerous other operations and he paid much attention to the subject of hernia, President Polk early in life being one of his subjects and one of those who were permanently cured by his skill. McDowell was not wealthy, his estate being estimated at from $40,000 to $50,000 at the time of his death. He expired at Danville, June 20, 1830. His disease was inflammatory fever, terminating his life on the fourteenth day of the attack, in the fifty-ninth year of his age,—and the remains of Kentucky's first great surgeon repose in the family burial ground of Governor Shelby, five miles from Danville. His tombstone, a plain slab of marble, bears the simple inscription of his name, Ephraim McDowell.[1]

Valentine Mott was the great surgeon of New York seventy-five years ago. He was born at Glen Cove, L. I., August 20, 1785. He was the first man to ligate the innominate artery on the living, in the year 1818. The patient was one Michael Bateman, a seaman from Massachusetts. The man lived twenty-five days after the operation and then died of recurring hemorrhages. In 1[illegible]7 he successfully tied the common illiac artery for aneurism, the second case ever so operated upon, and Mott's patient was the first to survive. The former and unsuccessful case was in the hands of another brilliant American surgeon, Dr. William Gibson, of Baltimore.

John Collins Warren is another surgeon connected with an epoch in American medicine. He was the son of John Warren, who founded the Harvard Medical School. It was he who allowed his name and position to stand sponsor for the first public use of ether anesthesia in the Massachusetts General Hospital, on Friday, October 16, 1846, when he operated upon one Gilbert Abbott for the removal of a congenital but superficial vascular tumor just below the jaw on the left side of the neck, the anesthetic being given by Dr. Morton, its discoverer.

Benjamin Winslow Dudley was born in Spottsylvania county, Virginia, on April 12, 1785, the son of a Baptist minister. The family moved into the neighborhood of Lexington, Ky., while the son was still a child. He began his professional studies in the family of an excellent general practitioner, Frederick Ridgely, and in 1804 went to Philadelphia where he graduated two years later. He went back to Lexington in 1806 and, after having made some money, loaded a flat-boat with a collection of sundries, and drifted down to New Orleans where he did some sharp trading, investing his all in flour, and sailed boldly for Gibraltar. He sold

---

1. The Kentucky State Medical Society erected a monument to McDowell at Danville which was dedicated May 14, 1879. Dr. L. S. McMurtry was chairman of the monument committee. McDowell's remains were removed to Danville and repose under the shadow of the monument. The remains of Mrs. McDowell were subsequently removed to the same spot.—*Note by the Editor.*

his cargo for a large sum at Gibraltar and Lisbon, left his vessel, pushed through Spain and reached Paris late in the same year. He remained nearly four years in Paris and some time in Great Britain, returning in 1814 to America and the wilds of Kentucky. He was noted as a great lithotomist. In the course of some forty years practice, he cut for stone two hundred and twenty times, the first hundred operations being without a death. In February, 1828, he published an article in the Transylvania Journal of Medicine on traumatic epilepsy, which created a genuine sensation. His conclusions were based on five cases on which he operated, with a resulting cure in three and marked benefit in the other two. He also described a new operation for hydrocele by excision of the sac, a method now commonly employed, also a series of articles on bandaging, a paper on fractures and one on stone in the bladder, these being the extent of his writings. In 1817 he induced the trustees of the Transylvania University to establish a medical department. In 1819 Dudley's best work began,—that excellent teaching that made him famous,—and his surgical practice grew rapidly, his fees were large and his early experience had made him a good man of business. He retired in 1850, after having built himself a comfortable country house, and died in 1870.

David Hosack was born in New York, August 31, 1769. He was well educated and began his medical studies in the office of Richard Bailey, a well-known surgeon of the day. His study of medicine at that time was very brief. Profiting by the written words of Morgan and Middleton, that a thorough preliminary education was essential, he went to Princeton and graduated a Bachelor of Arts in 1789. Returning to New York, he entered seriously upon the study of medicine, under former teachers of the old Columbia school. After one year he went to Philadelphia and graduated an M.D. in 1791 at the age of twenty-two. He established himself at Alexandria, in Virginia, but after a short stay he returned to New York and, observing the distinction our citizens made between those physicians who had been educated at home and those who had received additional instruction from the universities of Europe, he went to Liverpool, Edinburgh and London, returning to this country in 1794. He was appointed professor of materia medica at the Columbia School when but twenty-eight years old and, on the founding of the College of Physicians and Surgeons, was appointed respectively to the chairs of botany and materia medica, surgery and midwifery, and finally, in 1811, on the reorganization and uniting of the two schools, he filled the chair on the theory and practice of physic and clinical medicine. Hosack was known as an eminent citizen as well as a popular teacher and a good doctor. At the age of sixty he retired to a comfortable country place on the Hudson and died in 1835 at the age of sixty-six years.

The two most conspicuous names in Boston medicine of the last century are those of Jackson and Bigelow. Jacob Bigelow was born February 27, 1787 and died January 10, 1879, being nearly ninety-two years old. He graduated from Harvard in 1806 and two years later went to Boston to attend the lectures of the medical professors, where he entered the office of John Graham and eked out a scanty income by teaching in the Boston Latin School. After one year he went to Philadelphia for the lectures of Rush, Weston, Physick, Barton, and Cox, and to obtain the doctorate degree. Returning to Boston the elder Jackson chose him as an associate in practice. In 1815 he was appointed lecturer on materia medica and botany and in 1817, at the age of thirty, his title was changed to professor, being the first Rumford professor. In 1820 he was appointed an editor of the first edition of the United States Pharmacopeia, and in 1825 he founded Mount Auburn Cemetery. Up to this time the dead of our cities had been buried in the city churchyards and vaults and the problem of their disposal was becoming an urgent and distressing one. Bigelow's proposition was the founding of an extra urban forest cemetery and after seven years of debate the cemetery at Mount Auburn was dedicated for public use. In 1835 he read an address on "Self limited diseases" before the Massachusetts Medical Society, whose effect was instantaneous and immense. Writing in 1880, Oliver Wendell Holmes says::

"This remarkable essay has probably had more influence on medical practice in America than any similar brief treatise, we might say than any work ever published in this country. Its suggestions were scattered abroad at the exact fertilizing moment when public opinion was matured enough for their reception."

In 1852 Bigelow delivered before the students of the Massachusetts Medical College, Boston, an essay on medical education in which he urged upon his students the great importance of a thorough scientific training. Among other things he defined the exact sciences and the speculative sciences. Among the latter he placed practical medicine, which he describes as—

"A science older than civilization, cultivated and honored by ages, powerful for good or for evil, progressive in its character but still unsettled in its principles, remunerative in fame and fortune to its successful cultivators and rich in the fruits of a good conscience to its honest votaries. Encumbered as it is with difficulty, fallacy and doubt, medicine yet constitutes one of the learned professions; its imperfections are lost sight of in the overwhelming importance of its objects; the living look to it for succor, the dying call on it for rescue,"—his final conclusions being, "that he is a great physician who above other men understands diagnosis."

Bigelow took a great interest in all things that tended to improve popular education and knowledge. In one of his addresses he says that:

"Education is the right of many and not the privilege of the few, that the conservatism that restricts education to the classics and what may be called esthetic culture is but the highest form of class selfishness, that such practices are not only in themselves vicious but tend to the lowering of the whole educational fabric, that the underlying thought in education is the teaching how to think and the meaning of study, and this much at least is due to the masses, that it is those things that tend most to the useful arts, to the alleviation of human suffering, to the broadening of the popular horizon for which we must all strive.

When 83 years old he went to California on a pleasure trip; he was blind for the last five years of his life, bedridden but with mind undimmed to the last.

Fearing that I have already exhausted your patience I can only mention some of those whose names shine bright in the galaxy of America's greatest medical men,—Nathaniel Chapman, of Virginia and Philadelphia, John W. Francis of New York, William Gibson of Baltimore and Philadelphia, James Jackson of Boston, Daniel Drake of Kentucky, Casper Wistar, the anatomist of Philadelphia, John Beale Davidge, surgeon, of Maryland, John Redman Cox of Philadelphia, John Eberle of Philadelphia, William Potts Dewees, the father of American Gynecology, William Beaumont, Hugh L. Hodge, Samuel D. Gross, Isaac Ray, Gurdon Buck, Alonzo Clark, Oliver Wendell Holmes, Austin Flint, James Marion Sims, Henry Jacob Bigelow,—these and others too numerous to mention, might occupy us for a full evening.

1072 Lovejoy Street.

